# Enhancing Transplanting Success in Restoration of Degraded Areas Using Peat-Free Substrates

**DOI:** 10.3390/plants14101450

**Published:** 2025-05-13

**Authors:** Silvia Traversari, Sara Di Lonardo, Simone Orsenigo, Daniele Massa, Beatrice Nesi, Lino Zubani, Sonia Cacini

**Affiliations:** 1 Research Institute on Terrestrial Ecosystems (IRET), National Research Council (CNR), Via G. Moruzzi 1, 56124 Pisa, Italy; silvia.traversari@cnr.it; 2 National Biodiversity Future Center (NBFC), Piazza Marina 61, 90133 Palermo, Italy; 3 Research Institute on Terrestrial Ecosystems (IRET), National Research Council (CNR), Via Madonna del Piano 10, 50019 Sesto Fiorentino, Italy; 4 Department of Earth and Environmental Sciences, University of Pavia, Via S. Epifanio 14, 27100 Pavia, Italy; simone.orsenigo@unipv.it; 5 CREA Research Centre for Vegetables and Ornamental Crops, Council for Agricultural Research and Economics, Via dei Fiori 8, 51017 Pescia, Italy; daniele.massa@crea.gov.it (D.M.); beatrice.nesi@crea.gov.it (B.N.); sonia.cacini@crea.gov.it (S.C.); 6 Flora Conservation s.r.l., Via Francana 500, 27100 Pavia, Italy; info@floraconservation.com

**Keywords:** native species, drought, sustainable growing media, ecological restoration, *Viburnum lantana* L.

## Abstract

Native plant species used for ecological restoration in urban and degraded areas are typically cultivated by ornamental and forestry nurseries. In the face of climate change, it is crucial to produce plants that can withstand transplant stress while promoting the use of sustainable materials, such as peat-free substrates. Replacing peat with locally sourced organic materials offers a promising strategy to enhance plant resilience to abiotic stress while improving sustainability. This study evaluated the effects of alternative growing media on the growth and post-transplant performance of *Viburnum lantana* L. under standard nursery conditions. Three substrate mixtures were tested: (i) peat:pumice 70:30 *v*:*v* (PP); (ii) coconut coir dust:pumice 70:30 *v*:*v* (CP); (iii) coconut coir dust:green compost 55:45 *v*:*v* (CGC). After one year in the nursery, half of the plants were sampled in late spring for biometric, eco-physiological, and nutrient analyses, while the remaining plants were transplanted into a degraded area providing only a single irrigation event during the trial. Approximately 100 days after transplant, biometric and eco-physiological parameters were assessed. Plants grown on CGC demonstrated the highest transplant success, while those grown on PP and CP exhibited greater leaf necrosis, with PP plants also showing significant defoliation. These findings highlight CGC as a viable and sustainable alternative to peat-based substrates, particularly for post-transplant survival in degraded areas prone to drought stress.

## 1. Introduction

The increasing need for peat-free substrates has gained significant attention in recent years, both in response to market trends and sustainability concerns. Peat extraction has long been criticized for its detrimental effects on carbon sequestration and biodiversity loss, prompting researchers and the horticultural industry to seek alternative growing media [1,2,3,4,5,6,7,8,9,10]. While substitutes such as coconut coir have gained widespread adoption due to their physical properties and availability, their overall environmental impact, particularly in terms of life cycle assessment (LCA), raises questions about their long-term viability [11]. The production and transport of coconut coir involves considerable energy consumption and greenhouse gas emissions, making it a less sustainable option than initially perceived. This highlights the importance of exploring more sustainable and locally available options to reduce dependence on materials with high ecological footprints. Compost, for instance, has emerged as a promising alternative due to its nutrient-rich composition, water retention capacity, and potential to enhance soil microbiota, with an expected increase in its usage to 500% by 2050 [8]. In parallel, the ecological restoration of degraded areas and the use of native plants have become crucial strategies for promoting biodiversity and ecosystem resilience and function [12,13]. Despite this, increasing efforts and multiple methodological challenges still persist in science, policy, and practice. Degraded ecosystems often suffer from soil depletion, reduced microbial activity, and increased vulnerability to extreme climatic conditions [14]. The use of native plants in ecological restoration projects has been shown to improve ecosystem stability and foster local biodiversity [15]. However, successful plant establishment depends on several factors, including the physical and chemical characteristics of the substrate [16,17,18]. Ensuring optimal eco-physiological responses in plants is essential for their survival in stress-prone environments, particularly in areas affected by drought, salinity, and nutrient depletion [19]. The choice of growing media plays a key role in enhancing plant performance, influencing factors such as water retention, nutrient availability, and overall adaptability to challenging conditions [20]. Recent studies have emphasized the role of organic amendments, including green compost, in improving soil structure and enhancing plant resilience [21]. This study aims to demonstrate how substrate selection, particularly through the use of sustainable and locally sourced materials such as green compost, can improve the eco-physiological responses of plants. Compost not only provides essential nutrients but also enhances microbial activity, which plays a crucial role in nutrient cycling and plant health [22,23]. By adopting environmentally friendly alternatives, it is possible to foster more resilient plant communities while reducing reliance on non-renewable or less sustainable resources. The results of this study may contribute to the growing body of knowledge supporting the transition towards sustainable substrate management in horticulture and ecological restoration.

## 2. Results

### 2.1. Cultivation Trial

The growth of *V. lantana* on the three tested substrates was continuously monitored. At the start of the trial (T0), plant height was comparable across all substrates (37.8 ± 7.1 cm). Table 1 presents data on plant height, collar diameter, total shoot biomass fresh weight (FW), shoot dry weight (DW)-to-FW ratio, leaf DW, specific leaf area (SLA), and root-to-shoot ratio, measured at the end of the cultivation trial (T3, i.e., 398 days after transplanting, DAT). No significant differences were observed in plant height, collar diameter, shoot FW, leaf DW, or root-to-shoot ratio among treatments. However, the shoot DW-to-FW ratio was significantly higher in PP compared to CGC, while CP plants showed intermediate values. Additionally, SLA was significantly higher in CGC treatments compared to both CP and PP treatments, with the lowest values recorded in PP, which was also significantly lower than CP.

**Figure 1:**
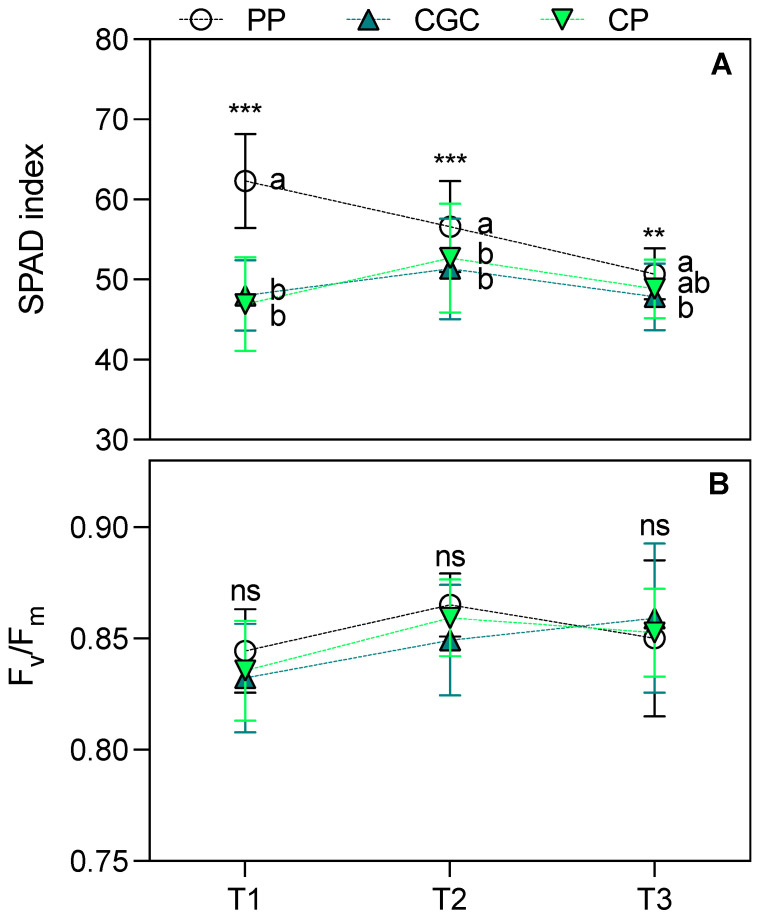
The SPAD index (**A**) and F_v_/F_m_ ratio (**B**) measured during the growing cycle at T1, T2, and T3 (74, 159, and 398 DAT, respectively). PP = peat:pumice; CP = coconut coir dust:pumice; CGC = coconut coir dust:green compost. The reported values have been calculated as the medium values of four replicates ± the standard deviation for each measurement point. Statistical analysis performed through one-way ANOVA at each sampling point: ns = not significant, or ** and *** = significant at *p* ≤ 0.01 and 0.001, respectively. Different letters for the same parameter indicate significant differences according to Tukey’s multiple-range test (*p* = 0.05).

**Figure 2:**
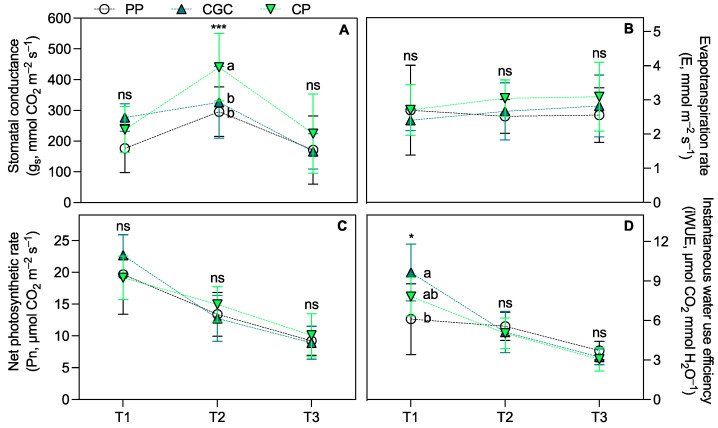
Stomatal conductance (**A**), evapotranspiration rate (**B**), net photosynthesic rate (**C**), and instantaneous water use efficiency (**D**) measured during the growing cycle at T1, T2, and T3 (74, 159, and 398 DAT, respectively). PP = peat:pumice; CP = coconut coir dust:pumice; CGC = coconut coir dust:green compost. The reported values have been calculated as the medium values of four replicates ± the standard deviation for each measurement point. Statistical analysis performed through one-way ANOVA at each sampling point: ns = not significant, or * and *** = significant at *p* ≤ 0.05 and 0.001, respectively. Different letters for the same parameter indicate significant differences according to Tukey’s multiple-range test (*p* = 0.05).

Regarding eco-physiological parameters, the SPAD index measured 74 and 159 DAT (T1 and T2, respectively) was significantly higher in plants grown on PP compared to those grown on CP and CGC. At T3, PP plants maintained higher values but only in comparison to CGC (Figure 1A). The maximal quantum yield of PSII (F_v_/F_m_; Figure 1B) showed no significant differences among treatments across all time points. Similarly, leaf transpiration (E) and net photosynthetic rate (Pn) (Figure 2B,C) did not differ significantly among treatments. However, CP plants exhibited the highest stomatal conductance (g_s_) at T2 compared to PP and CGC (Figure 2A). Additionally, PP plants showed the lowest leaf instantaneous water use efficiency (iWUE) at T1 compared to CGC (Figure 2D).

**Figure 3:**
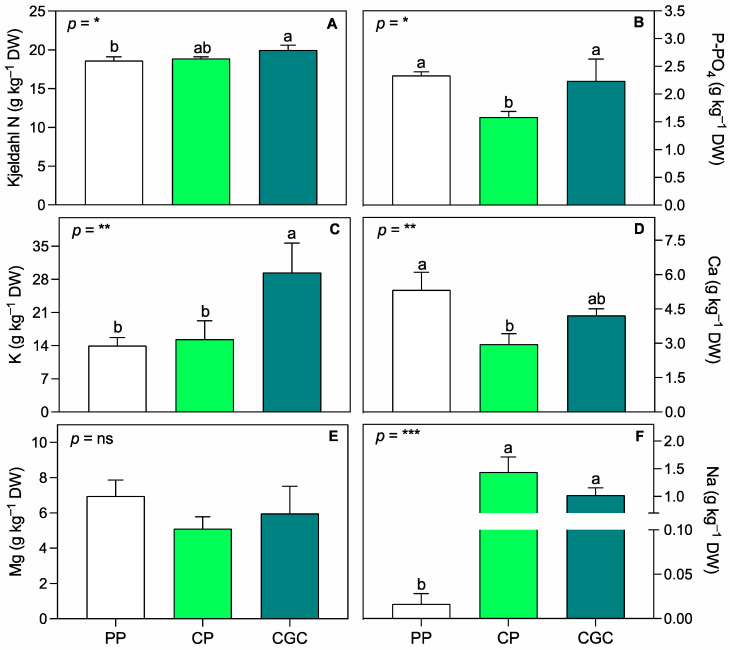
Leaf nutrient concentration, i.e., Kjeldahl nitrogen (**A**), phosphorus P-PO_4_ (**B**), potassium (**C**), calcium (**D**), magnesium (**E**), and sodium (**F**), assessed after one year of cultivation at T3 (398 DAT). PP = peat:pumice; CP = coconut coir dust:pumice; CGC = coconut coir dust:green compost. The reported values have been calculated as the medium values of four replicates + the standard deviation. Statistical analysis performed through one-way ANOVA: ns = not significant, or *, **, and *** = significant at *p* ≤ 0.05, 0.01, and 0.001, respectively. Different letters for the same parameter indicate significant differences according to Tukey’s multiple-range test (*p* = 0.05).

In terms of leaf tissue elemental composition, Kjeldahl N was significantly higher in CGC than in PP plants (Figure 3A). Conversely, P-PO_4_ was, on average, 30% lower in CP than in PP and CGC plants, while K was, on average, 50% higher in CGC compared to both PP and CP plants. As a meso-nutrient, Ca was significantly higher in PP than in CP plants, while Mg did not show significant differences among treatments. Sodium levels in leaf tissue were higher in both peat-free substrate treatments compared to PP.

### 2.2. Plant Performances After Transplanting Trial in a Degraded Area

After transplantation, biometric and eco-physiological parameters were evaluated after 98 days (T4). Plants grown on CGC exhibited significantly higher leaf FW compared to PP plants (+45.1%), where severe defoliation was observed. However, no significant differences were found in shoot DW and shoot DW percentage among treatments. Notably, PP and CP treatments showed a significantly higher percentage of leaf necrosis (+69% on average) compared to CGC. Additionally, PP plants exhibited a significantly higher stem DW-to-shoot DW ratio (Table 2).

**Figure 4:**
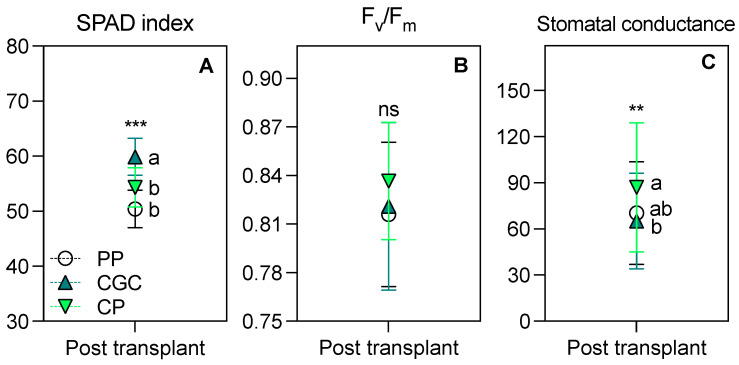
The SPAD index (**A**), F_v_/F_m_ ratio (**B**), and stomatal conductance (g_s_, mmol CO_2_ m^−2^ s^−1^, **C**) measured at 98 days from transplanting in a degraded area. The reported values have been calculated as the medium values of four replicates ± the standard deviation. Statistical analysis performed through one-way ANOVA: ns = not significant, or ** and *** = significant at *p* ≤ 0.01 and 0.001, respectively. Different letters for the same parameter indicate significant differences according to Tukey’s multiple-range test (*p* = 0.05). PP = peat:pumice; CP = coconut coir dust:pumice; CGC = coconut coir dust:green compost.

As shown in Figure 4, plants grown on CGC had a significantly higher SPAD index compared to the other two treatments. Furthermore, CGC plants exhibited the lowest g_s_, though the difference was significant only compared to PP. No significant differences were observed in the F_v_/F_m_ ratio among treatments.

## 3. Discussion

The use of peat-free growing media is an increasingly widespread practice in horticulture, including for ornamental plants. Numerous studies have demonstrated that peat alternatives can achieve comparable high-quality standards [24,25,26]. In this study, plants grown on two peat-free substrates composed primarily of coconut coir dust and green compost exhibited growth and quality similar to those cultivated on peat-based media. The only notable difference was a slightly lower DW accumulation in the CGC substrate (approximately 6% lower than in PP and CP). Additionally, SLA varied significantly among treatments, following the trend PP < CP < CGC. This parameter is a key morphological trait influencing leaf thickness and width, both of which are desirable attributes in ornamental plants. It is also widely recognized as an indicator of photosynthetic capacity and drought tolerance [27,28,29]. While drought resistance is often associated with reduced SLA, some ornamental species do not exhibit negative effects under water deficiency, maintaining a high absorption surface per unit of leaf biomass [30]. During the nursery phase, plants grown on CGC and CP substrates had a lower SPAD index than on PP at T1 and T2, with CGC plants maintaining slightly lower SPAD values at T3 as well. Since the SPAD index correlates with chlorophyll content, this result is not entirely surprising, considering the well-documented negative relationship between SLA values and the SPAD index [31]. Importantly, no significant differences were found in the F_v_/F_m_ ratio, which remained above 0.83 across all treatments. This indicates consistently high intrinsic efficiency of the PSII and the absence of stress-induced photoinhibition [32,33]. Gas exchange analysis during the nursery phase revealed no significant differences among treatments, except for higher g_s_ values in CP at T2, prior to winter dormancy. This finding can be correlated to tissue nutrient content, with CP showing a reduced value of Ca concentration, and thus to probably less efficient stomatal regulation [34,35], as further discussed below. Notably, plants grown on CGC exhibited higher iWUE during July (T1), one of the hottest months in the Mediterranean region, which is characterized by high evapotranspiration rates [36]. Despite this, g_s_, Pn, and E values in CGC plants remained comparable to those of other treatments. The iWUE is a key indicator of plant drought stress response, with higher values indicating greater water use efficiency and resilience under limited water availability [37,38,39]. The specific physiological responses aligned with nutrient leaf concentrations observed at the end of the nursery growing phase, which in turn reflected the substrate composition (Table S1) and agreed with other studies conducted on perennial herbaceous plants on the same mixes [40,41]. CGC-grown plants exhibited higher Kjeldahl N and K levels compared to PP, in agreement with the higher concentrations of these nutrients in the substrate. The increased N and K content likely contributed to enhance photosynthetic performance, as both nutrients play essential roles in mesophyll conductance and the carboxylation capacity of Ribulose 1,5-bisphosphate [42]. Conversely, CP-grown plants had lower leaf P-PO_4_ and Ca concentrations compared to both PP and CGC, suggesting that plants grown on the CP substrate experienced a greater physiological stress during the summer period. However, the measured values remained within or above the reference threshold for these elements [43,44], indicating no severe deficiencies. Considering the initial composition of the substrates, the higher Na observed in plants grown in peat-free treatments was expected. However, no adverse effects related to Na toxicity, such as strong inhibition of K, Mg, or Ca uptake or leaf necrosis, were observed [45].

Nursery agronomic practices have a significant impact on plant survival and growth after transplantation [46]. The performance of plants in the nursery phase was consistent with the observations made after transplantation in a degraded area. Plants grown on the CGC substrate, which performed best in nursery conditions, also showed the strongest post-transplantation response, outperforming those grown on PP. Then, CP-grown plants showed an intermediate behavior. Plants grown on CGC exhibited superior vegetative growth compared to PP, partly due to a significant defoliation. However, considering the higher percentage of necrotic leaf area observed in PP and CP plants compared to CGC, the intermediate response of CP plants in terms of vegetative growth needs to be weighted. The greater response of CGC plants was further highlighted by the eco-physiological assessment, where plants grown on CGC maintained the highest SPAD index values. Moreover, CGC plants demonstrated superior drought resistance, as evidenced by a more efficient stomatal regulation (i.e., g_s_ values similar to or lower than PP before and after transplantation), thus protecting plants from dehydration and xylem disfunction and ensuring high water use efficiency, as also observed in many species [47,48,49,50]. Interestingly, no significant differences were found in the F_v_/F_m_ ratio among treatments, suggesting that photosystem II (PSII) function remained unaffected. This might seem contradictory, given the lower post-transplant growth and greater leaf necrosis observed in PP and CP treatments. However, it is important to note that the F_v_/F_m_ ratio was assessed on the last fully expanded leaves, whereas necrotic areas were primarily found on older foliage that had developed three months post-transplantation, i.e., at the end of summer stress period. By this time, environmental and climatic conditions—particularly temperature and humidity—had already begun to improve.

In conclusion, our findings suggest that the use of peat-free substrates in native plant cultivation can enhance post-transplant performance and improve plant survival rates in ecological restoration projects, ultimately supporting more sustainable reforestation and landscape rehabilitation efforts. Green compost emerges as a particularly promising alternative due to its local availability, lower environmental impact, and ethical advantages over coconut-based products [17], as well as its well-documented role as a growth promoter [51,52]. In our experimental conditions, CGC promoted biomass production and enhanced plant eco-physiological responses and resilience to abiotic stress, confirming its suitability for use in degraded environments. CP may also represent a viable alternative to peat-based substrates; however, plants grown on it exhibited slower adaptative responses, similar to those grown on PP, and did not show a clear performance advantage.

## 4. Materials and Methods

### 4.1. Cultivation Trial: Growing Condition and Experimental Design

A one-year cultivation trial was carried out to evaluate the effects of three different substrates on the Southern European thermophilus and deciduous shrub *Viburnum lantana* L. (*Viburnaceae*) under standard nursery conditions. The experiment took place at an open-field nursery, Flora Conservation s.r.l., in Pavia, Italy (latitude 45.17° N, longitude 9.19° E, altitude 77 m), which is equipped for soilless cultivation. The plant material consisted of one-year-old seedlings transplanted into 4 L pots. The three tested substrates were as follows: (i) peat:pumice 70:30 *v*:*v*, used as control (PP); (ii) coconut coir dust:pumice 70:30 *v*:*v* (CP); (iii) coconut coir dust:green compost 55:45 *v*:*v* (CGC). These substrates had been previously characterized for their chemical and physical properties and tested in earlier experiments on perennial herbaceous species and shrubs [40,41]. A brief description of their chemical and physical proprieties is reported in Table S1. The substrates were appropriately mixed and supplied by TERFLOR s.r.l. (Capriolo, BS, Italy), then enriched with a mineral fertilizer at a dosage of 1.0 kg m^−3^ (PG MIXTM, 14-16-18), a controlled release fertilizer at a dosage of 1.5 kg m^−3^ (Osmocote^®^ Exact Standard 8–9 months, 15-9-11 + 2MgO + TE), and 5 kg m^−3^ calcium carbonate in the control substrate. The trial began on 28 April 2017, with pot transplanting, and lasted until 30 May 2018. Each treatment included 40 plants arranged in four replicates using a randomized block design (n = 120 plants). Irrigation was provided by a sprinkler irrigation system controlled by a timer, adjusted according to weather conditions. At the beginning of the second growing season (27 April 2018), all plants were fertilized with a top-dress fertilizer (Osmocote^®^ Topdress 5–6 months, 19-6-11 + 2MgO + 0.5Fe) at a rate of 4 kg m^−3^ per pot, according to the standard grower agronomic practices to guarantee optimal vegetative recovery.

During the study period, the average minimum and maximum temperatures were 10.4 °C and 19.4 °C, respectively, with a mean relative humidity of 68%. Climatic data were collected from a local weather station in Pavia.

### 4.2. Transplanting Trial in a Degraded Area

Following the nursery phase, a transplanting trial was conducted after one year to assess plant performance in a degraded environment. A total of 12 plants per treatment were randomly assigned to four replicates in a completely randomized design, simulating a hedge formation with 50 cm spacing between plants. The transplanting took place on May 31, 2018, in a degraded area within “Parco Regionale Lombardo della Valle del Ticino” (Pavia, Italy), near the Flora Conservation s.r.l. nursery in a homogeneous area. The soil was classified as sandy loam, with a sub-alkaline pH, and was not amended with fertilizers (organic or inorganic) prior to transplanting. The site lacked an irrigation system, and plants were only irrigated once immediately after transplanting. The trial lasted until 6 September, 2018 (98 days). Weather conditions during this period were characterized by a dry summer, with only 12 days recording more than 10 mm of rainfall. The total precipitation amounted to 335 mm, while the average minimum and maximum temperatures were 19.4 °C and 29.8 °C, respectively, with a mean relative humidity of 64%.

### 4.3. Biometric, Eco-Physiological, and Nutrient Analyses

During the one-year cultivation trial in the nursery, plant growth and eco-physiological parameters were monitored at key developmental stages: (i) T0 (28 April 2017), transplanting; (ii) T1 (74 DAT, 10 July 2017), mid-summer vegetative phase; (iii) T2 (159 DAT, 3 October 2017), pre-winter dormancy phase; (iv) T3 (398 DAT, 30 May 2018), post-spring vegetative growth phase. Then, plants were monitored 98 d after transplantation in soil of a degraded area (T4; 6 September 2018).

Plant height and collar diameter were monitored at T0, T1, T2, and T3. Eco-physiological parameters were collected at T1, T2, and T3 as follows: (i) chlorophyll *a* (Chl *a*) fluorescence was measured on 20 min dark-adapted leaves and recorded as the F_v_/F_m_ ratio (maximal quantum yield of photosystem II, PSII) from 09:30 to 13:00 on 6 plants per replicate using a Pulse–Amplitude–Modulation fluorometer (MINI-PAM, Heine Walz, Germany); (ii) the SPAD index was measured on 8 fully expanded leaves per plant (10 plants per replicate) using a SPAD-502 (Konica Minolta Optics, 2970 Ishikawa-machi, Hachioji, Tokyo, Japan); (iii) stomatal conductance (g_s_), net photosynthetic rate (Pn), and leaf transpiration (E) were measured on 4 plants per replicate from 09:30 to 13:00 using a portable gas exchange system CIRAS-2 (PP Systems, Amesbury, MA, USA). The leaf instantaneous water use efficiency (iWUE, expressed as μmol CO_2_ mmol H_2_O^−1^) was then calculated as the Pn-on-E ratio [53,54].

At T3, plants were harvested to assess fresh (FW) and dry (DW) total biomass, root DW, leaf area (LA), and specific leaf area (SLA). The two last parameters were determined on fresh leaves using the WinDIAS Image Analysis System (Delta-T Devices, Cambridge, UK). The DW was obtained by oven-drying samples at 75 °C until a constant weight was reached. Dry leaf samples were powdered using a mill to conduct leaf nutrient analysis. Total Kjeldahl N was determined using the Kjeldahl method after phospho-sulfuric acid digestion [55]. For macro and microelement determination, 200 mg of shoot samples was wet-digested in a mixture of nitric and perchloric acids (HNO_3_:HClO_4_ 5:2 *v*:*v*) at 230 °C for 1 h. Atomic absorption spectrometry was used to quantify Ca, K, Mg, and Na, while a spectrophotometer (Evolution™ 300 UV–Vis Spectrophotometer, Thermo Fisher Scientific Inc., Waltham, MA, USA) was employed for P determination using the molybdenum blue method.

At the end of the field transplanting trial in the degraded area (T4), plant growth and eco-physiological performances were evaluated on all plants. Aboveground (leaves and stems) FW and DW, obtained by cutting plants at a uniform height of 30 cm, LA and the percentage of necrotic leaf area, Chl *a* fluorescence (F_v_/F_m_ ratio), and the SPAD index were assessed by using the methods previously described. Among gas exchange parameters, g_s_ was measured on 12 leaves per replicate using an AP4 portable leaf porometer (Delta-T Devices, UK).

### 4.4. Statistical Analysis

A one-way analysis of variance (ANOVA) was performed to assess the effect of the different substrates on biometric, eco-physiological, and nutrient concentration parameters during both the nursery trial and post-transplant phase in the degraded area. Mean comparisons were conducted using Tukey’s Honest Significant Difference (HSD) test. The data are presented as the mean ± standard deviation (SD) unless otherwise specified. Statistical and graphical analysis were conducted using Statgraphics Centurion XV (Statpoint Technologies, Inc., Warrenton, VA, USA) and Prism 10 (GraphPad Software, Inc., La Jolla, CA, USA).

## Figures and Tables

**Table 1:** Plant height, collar diameter, total shoot fresh weight (FW), shoot dry weight (DW)-to-FW ratio, leaf DW, specific leaf area (SLA), and root-to-shoot ratio measured at the end of the growing cycle, T3 (i.e., 398 DAT). PP = peat:pumice; CP = coconut coir dust:pumice; CGC = coconut coir dust:green compost.

Substrate	Plant Height	Collar Diameter	Shoot FW	Shoot DW FW^−1^	Leaf DW	SLA	Root/Shoot
	cm Plant^−1^	mm Plant^−1^	g Plant^−1^	%	g Plant^−1^	cm^2^ g DW^−1^	
PP	90.0 ± 17.9	22.1 ± 5.5	365.4 ± 86.2	35.9 ± 1.5 a	59.8 ± 16.7	81.1 ± 11.6 c	0.24 ± 0.05
CP	90.5 ± 15.3	19.3 ± 4.7	423.7 ± 154.5	35.3 ± 3.0 ab	70.4 ± 27.3	113.0 ± 13.3 b	0.27 ± 0.06
CGC	92.8 ± 17.9	18.4 ± 4.0	403.6 ± 62.3	33.4 ± 1.5 b	59.1 ± 16.0	141.8 ± 28.4 a	0.32 ± 0.13
ANOVA	ns	ns	ns	*	ns	***	ns

* Values are the means of three replicates ± the standard deviation. Statistical analysis performed through one-way ANOVA: ns = not significant, or * and *** = significant at *p* ≤ 0.05 and 0.001, respectively. Different letters for the same parameter indicate significant differences according to Tukey’s multiple-range test (*p* = 0.05).

**Table 2:** Leaf fresh weight (FW), shoot dry weight (DW), shoot DW-to-FW ratio, necrotic leaf area (LA)-to-total LA ratio, and stem DW-to-shoot DW ratio measured at 98 days from transplanting in a degraded area. PP = peat:pumice; CP = coconut coir dust:pumice; CGC = coconut coir dust:green compost.

Substrate	Leaf FW	Shoot DW	Shoot DW FW^−1^	Necrotic LA	Stem DW Shoot DW^−1^
	g Plant^−1^	g Plant^−1^	% Plant^−1^	% Plant^−1^	% Plant^−1^
PP	36.6 ± 18.4 b	80.3 ± 20.9	46.1 ± 3.0	14.6 ± 10.7 a	83.1 ± 12.0 a
CP	56.8 ± 20.1 ab	98.1 ± 35.4	44.7 ± 3.7	16.6 ± 8.9 a	76.9 ± 8.7 ab
CGC	66.6 ± 21.6 a	89.4 ± 20.2	44.9 ± 2.9	4.8 ± 1.6 b	72.2 ± 7.9 b
ANOVA	*	ns	ns	***	**

* Values are the means of four replicates ± the standard deviation. Statistical analysis performed through one-way ANOVA: ns = not significant, or *, **, and *** = significant at *p* ≤ 0.05, 0.01, and 0.001, respectively. Different letters for the same parameter indicate significant differences according to Tukey’s multiple-range test (*p* = 0.05).

## Data Availability

The dataset is available on request from the authors.

## Supplementary Materials

The following supporting information can be downloaded at https://www.mdpi.com/article/10.3390/plants14101450/s1: Table S1: Substrate parameters.
