# Supplementary material for: Enhancing Transplanting Success in Restoration of Degraded Areas Using Peat-Free Substrates
Source: Plants (Basel). 2025 May 13;14(10):1450. doi: 10.3390/plants14101450 (PMC12114766; doi:10.3390/plants14101450)
Supplement: Supplementary file 1 [file plants-14-01450-s001.zip › plants-3603059-supplementary.pdf]

Table S1. Chemical and physical properties of the three tested substrates (PP = peat:pumice. CP = coconut coir dust:pumice. CGC = coconut coir dust:green compost). BD = bulk density. TP = total porosity. W -1kPa = water holding capacity. AWC = available water content. AFP = air-filled porosity. n.d. = not detectable. Reported values have been calculated as mean values of three replicates. ANOVA analysis: ns = not significant or *, **, *** = significant at $P \leq 0.05$, 0.01 and 0.001, respectively; different letters for the same parameter indicate significantly different values (HSD test, $P < 0.05$). Data reported in [35-36].

Parameters	PP	CP	CGC	ANOVA
pH	6.7 c	7.1 b	7.8 a	***
EC (μS cm^{-1})	114.1 c	235.4 b	777.7 a	***
N-NH_4 (mg L^{-1})	7.4 b	12.5 b	40.7 a	***
N-NO_3 (mg L^{-1})	10.7 b	9.3 b	59.3 a	***
P-PO_4 (mg L^{-1})	n.d.	n.d.	5.60 a	***
K (mg L^{-1})	46.7 c	156.7 b	1015.0 a	***
Ca (mg L^{-1})	35.5 b	32.0 b	82.0 a	***
Mg (mg L^{-1})	8.8 b	10.1 b	21.9 a	***
Fe (mg L^{-1})	0.55	2.33	1.98	ns
Mn (mg L^{-1})	0.03 c	0.05 c	0.15 ab	***
Cu (mg L^{-1})	0.07 b	0.07 b	0.30 a	***
Zn (mg L^{-1})	0.10 b	0.10 b	0.35 a	***
B (mg L^{-1})	0.17 b	0.43 ab	0.65 a	*
**Physical properties**				
BD (g cm^{-3})	0.26 b	0.28 a	0.21 c	***
TP % ($v\ v^{-1}$)	86.8	85.5	87.2	ns
W -1kPa % ($v\ v^{-1}$)	59.2 a	53.6 ab	58.3 a	*
AWC % ($v\ v^{-1}$)	18.3	18.3	17.8	ns
AFP % ($v\ v^{-1}$)	27.6	32.4	28.9	ns
